# Hospital Performance, the Local Economy, and the Local Workforce: Findings from a US National Longitudinal Study

**DOI:** 10.1371/journal.pmed.1000297

**Published:** 2010-06-29

**Authors:** Jan Blustein, William B. Borden, Melissa Valentine

**Affiliations:** 1 Robert F. Wagner Graduate School and Division of General Medicine, NYU Medical School, New York University, New York, New York, United States of America; 2 Division of Cardiology, Department of Medicine and Department of Public Health, Weill Cornell Medical College, Cornell University, New York, New York, United States of America; 3 Program in Health Policy, Harvard University, Boston, Massachusetts, United States of America; Edinburgh University, United Kingdom

## Abstract

**Background:**

Pay-for-performance is an increasingly popular approach to improving health care quality, and the US government will soon implement pay-for-performance in hospitals nationwide. Yet hospital capacity to perform (and improve performance) likely depends on local resources. In this study, we quantify the association between hospital performance and local economic and human resources, and describe possible implications of pay-for-performance for socioeconomic equity.

**Methods and Findings:**

We applied county-level measures of local economic and workforce resources to a national sample of US hospitals (*n* = 2,705), during the period 2004–2007. We analyzed performance for two common cardiac conditions (acute myocardial infarction [AMI] and heart failure [HF]), using process-of-care measures from the Hospital Quality Alliance [HQA], and isolated temporal trends and the contributions of individual resource dimensions on performance, using multivariable mixed models. Performance scores were translated into net scores for hospitals using the Performance Assessment Model, which has been suggested as a basis for reimbursement under Medicare's “Value-Based Purchasing” program. Our analyses showed that hospital performance is substantially associated with local economic and workforce resources. For example, for HF in 2004, hospitals located in counties with longstanding poverty had mean HQA composite scores of 73.0, compared with a mean of 84.1 for hospitals in counties without longstanding poverty (*p*<0.001). Hospitals located in counties in the lowest quartile with respect to college graduates in the workforce had mean HQA composite scores of 76.7, compared with a mean of 86.2 for hospitals in the highest quartile (*p*<0.001). Performance on AMI measures showed similar patterns. Performance improved generally over the study period. Nevertheless, by 2007—4 years after public reporting began—hospitals in locationally disadvantaged areas still lagged behind their locationally advantaged counterparts. This lag translated into substantially lower net scores under the Performance Assessment Model for hospital reimbursement.

**Conclusions:**

Hospital performance on clinical process measures is associated with the quantity and quality of local economic and human resources. Medicare's hospital pay-for-performance program may exacerbate inequalities across regions, if implemented as currently proposed. Policymakers in the US and beyond may need to take into consideration the balance between greater efficiency through pay-for-performance and socioeconomic equity.

*Please see later in the article for the Editors' Summary*

## Abstract 2

Blustein and colleagues examine the associations between changes in hospital performance and their local economic resources. Locationally disadvantaged hospitals perform poorly on key indicators, raising concerns that pay-for-performance models may not reduce inequality.

## Editors' Summary

**Background:**

These days, many people are rewarded for working hard and efficiently by being given bonuses when they reach preset performance targets. With a rapidly aging population and rising health care costs, policy makers in many developed countries are considering ways of maximizing value for money, including rewarding health care providers when they meet targets, under “pay-for-performance.” In the UK, for example, a major pay-for-performance initiative—the Quality and Outcomes Framework—began in 2004. All the country's general practices (primary health care facilities that deal with all medical ailments) now detail their achievements in terms of numerous clinical quality indicators for common chronic conditions (for example, the regularity of blood sugar checks for people with diabetes). They are then rewarded on the basis of these results.

**Why Was This Study Done?:**

In the US, the government is poised to implement a nationwide pay-for-performance program in hospitals within Medicare, the government program that provides health insurance to Americans aged 65 years or older, as well as people with disabilities. However, some observers are concerned about the effect that the proposed pay-for-performance program might have on the distribution of health care resources in the US. Pay-for-performance assumes that health care providers have the economic and human resources that they need to perform or to improve their performance. But, if a hospital's capacity to perform depends on local resources, payment based on performance might worsen existing health care inequalities because hospitals in under-resourced areas might lose funds to hospitals in more affluent regions. In other words, the government might act as a reverse Robin Hood, taking from the poor and giving to the rich. In this study, the researchers examine the association between hospital performance and local economic and human resources, to explore whether this scenario is a plausible result of the pending change in US hospital reimbursement.

**What Did the Researchers Do and Find?:**

US hospitals have voluntarily reported their performance on indicators of clinical care (“process-of-care measures”) for acute myocardial infarction (AMI, heart attack), heart failure (HF), and pneumonia under the Hospital Quality Alliance (HQA) program since 2004. The researchers identified 2,705 hospitals that had fully reported process-of-care measures for AMI and HF in both 2004 and 2007. They then used the “Performance Assessment Model” (a methodology developed by the US Centers for Medicare and Medicaid Services to score hospital performance) to calculate scores for each hospital. Finally, they looked for associations between these scores and measures of the hospital's local economic and human resources such as population poverty levels and the percentage of college graduates in the workforce. Hospital performance was associated with local and economic workforce capacity, they report. Thus, hospitals in counties with longstanding poverty had lower average performance scores for HF and AMI than hospitals in affluent counties. Similarly, hospitals in counties with a low percentage of college graduates in the workforce had lower average performance scores than hospitals in counties where more of the workforce had been to college. Finally, although performance improved generally over the study period, hospitals in disadvantaged areas still lagged behind hospitals in advantaged areas in 2007.

**What Do These Findings Mean?:**

These findings indicate that hospital performance (as measured by the clinical process measures considered here) is associated with the quantity and quality of local human and economic resources. Thus, the proposed Medicare hospital pay-for-performance program may exacerbate existing US health care inequalities by leading to the transfer of funds from hospitals in disadvantaged locations to those in advantaged locations. Although further studies are needed to confirm this conclusion, these findings have important implications for pay-for-performance programs in health care. They suggest that US policy makers may need to modify how they measure performance improvement—the current Performance Assessment Model gives hospitals that start from a low baseline less credit for improvements than those that start from a high baseline. This works against hospitals in disadvantaged locations, which start at a low baseline. Second and more generally, they suggest that there may be a tension between the efficiency goals of pay-for-performance and other equity goals of health care systems. In a world where resources vary across regions, the expectation that regions can perform equally may not be realistic.

**Additional Information:**

Please access these Web sites via the online version of this summary at http://dx.doi.org/10.1371/journal.pmed.1000297.

- KaiserEDU.org is an online resource for learning about the US health care system. It includes educational modules on such topics as the Medicare program and efforts to improve the quality of care
- The Hospital Quality Alliance provides information on the quality of care in US hospitals
- Information about the UK National Health Service Quality and Outcomes Framework pay-for-performance initiative for general practice surgeries is available

## Introduction

Pay-for-performance is an important market-based approach to improving health care quality. During the past decade, the approach has been adopted widely, by health systems in the UK [1], Australia [2], and Taiwan [3], among others. Pay-for-performance has also been used in the US, but in a piecemeal fashion, in some states, by some insurance firms, for some health care providers [4]. Now a unified effort is underway, with the US government poised to implement pay-for-performance nationwide within its Medicare health insurance program [5],[6]. As the provider of near-universal health insurance to Americans age 65 y and older, Medicare is a powerful driver of US health care policy. For example, Medicare was the innovator in the introduction of hospital prospective payment under the Diagnosis Related Groups [DRGs] program [7]. That payment reform was in turn adopted in the private health insurance sector, and is the standard throughout the US today. Medicare reforms also resonate internationally, as evidenced by the widespread implementation of the DRG case mix approach in 25 nations worldwide [8].

Pay-for-performance assumes that providers have adequate economic and human resources to perform, or improve their performance, within a short time frame. Yet the prevailing distribution of resources in the US health care system makes it difficult for some providers to operate effectively as it is [9]. Payment based on performance may worsen inequalities, as hospitals in underresourced areas lose funds to their better-off counterparts, with the government acting as a sort of “reverse Robin Hood.”

This scenario is not entirely far-fetched. In the US, hospital revenues are largely derived from a mix of private and public health insurance payments, which vary with local socioeconomic conditions [9]. Strong finances give hospitals the opportunity to invest in quality improvement [10]. Hospitals also draw on local human resources. Arguably the most important of these is clinical staff. But not all facilities have access to a high quality talent pool. To date, much research and policy attention has been directed toward attracting physicians, nurses, pharmacists, and other clinicians to areas that would be otherwise underserved, because of local poverty, limited spousal employment opportunities, and sub-par schools [11]–[13].

Moreover, the US—and the world at large—is increasingly segregated, both economically and in terms of educational level [14]–[16]. Demographers in the US have noted a growing concentration of college-educated people in a relatively small subset of geographical areas [16]. This “regional concentration of human capital” has translated into higher productivity in places with more educated workforces, and a decline of economies in areas where this advantage is lacking [17],[18]. Although there is evidence that clinical outcomes vary by geographical area [19], little research has explored the impact of the regional concentration of wealth and human capital on health care.

In this study, we examine the association between local resources and hospital performance, seeking to understand the potential redistribution of funds under an important pending change in hospital reimbursement. We also explore the implications of our findings for health systems beyond the US, as pay-for-performance expands worldwide.

## Methods

### Setting

The US Medicare program is administered through the Centers for Medicare and Medicaid Services (CMS), a federal governmental agency. Over the past decade, the CMS has piloted pay-for-performance in a variety of settings [6]. Under the agency's ambitious “Value-Based Purchasing (VBP) Initiative,” the first wave of national implementation is slated to take place in hospitals, which will have a portion of their revenues withheld and then returned, conditional on their ability to meet quality targets [6]. Later, the approach will be extended to payment for other types of providers, including physicians, nursing homes, and home health agencies [6].

Groundwork for hospital pay-for-performance was laid when, in 2004, the agency called for hospitals to voluntarily report their performance on process-of-care measures for three clinical conditions (acute myocardial infarction [AMI], heart failure [HF], and pneumonia). Shortly thereafter, the agency began providing financial incentives for reporting, under so called “pay-for-reporting” [20]. The transition was to have been made to hospital pay-for-performance in 2009, but with the change in administration and focus on health care reform, that effort was temporarily suspended. Nonetheless, pay-for-performance enjoys a high degree of support in the agency, the Congress, the hospital industry, and the Obama administration, and is widely expected to be implemented, with the recent passage of health care reform [21].

### Data Sources and Sample

The performance data used in this study were derived during Medicare's voluntary reporting period from 2004–2007. Hospitals were eligible for inclusion in the study if they were located in the 50 United States, and voluntarily reported to the Medicare program under the Hospital Quality Alliance [HQA] program during the period. We merged the HQA process-of-care data (which are publicly posted on the program's Hospital Compare website [22]) with data on hospital characteristics and finances from the Medicare Cost Reports [23]. These data were merged with county-level information from the Health Resources and Services Administration's Area Resource File [24].

We used a dataset of institutions reporting at least some HQA data for at least 1 y during the study period. Because a goal of the study was to assess change in all measures over time, we limited the study sample to those hospitals reporting on all seven measures in both 2004 and 2007 (*n* = 2,705; see below).

### Measures

#### Composite HQA performance score

We used clinical process-of-care measures for AMI and HF, two of the three conditions for which process measures were collected under the HQA throughout the study period. We do not present findings on the third condition (pneumonia), because the initial measures for that condition were controversial and were modified during the study period [25]. However, findings for the pneumonia measures were qualitatively similar to those reported here for AMI and HF, albeit with somewhat attenuated impacts.

Detailed standards for these measures are published elsewhere [26]. Consistent with previous research [27],[28], we selected the following individual measures in developing composite scores: AMI (aspirin on admission, aspirin at discharge, angiotensin converting enzyme [ACE] inhibitor for left ventricular dysfunction, beta-blocker on admission, beta-blocker at discharge); HF (assessment of left ventricular function, ACE inhibitor for left ventricular dysfunction). These are process measures that can be successfully met by physician order or chart notation, including documentation of a contraindication. We excluded measures of the delivery of cognitive services such as smoking cessation counseling that may be sensitive to patient characteristics [29]. For each year, for each of the two conditions, we computed a single weighted average “composite” score, following a standard methodology [30], which assigned each hospital a score ranging from 0 to 100, reflecting the mean hospital performance on a patient receiving the processes of care for which s/he was eligible, for that condition.

#### Locational resources

We characterized hospital locational resources at the county level across a set of dimensions. Local economic conditions were measured in two ways. First, chronicity of local poverty was assessed using a modified version of a metric developed by the US Department of Agriculture's Economic Research Service [ERS], which identifies counties with respect to their population poverty levels over the past four decennial census periods (1970–2000). “Persistently poor” counties had >20% of their population living in poverty in all four of those census years; “intermittently poor” counties met the >20% criterion during at least one census year; and “never poor” counties never met the >20% poverty level during any of the census years. The current health of the local economy was summarized by the local unemployment rate according to the 2000 census, using the ERS cut point for “high” unemployment (<65% of residents 21–64 y old employed).

Three measures reflected the availability and characteristics of the local workforce. Availability of health professionals was measured using the federal government's Health Professional Shortage Area (Primary Care) (HPSA) designation [31]. The HPSA designation applied to the whole county, a portion of the county, or to no portion of the county. The education level of the local workforce was measured in two ways. The ERS's “low education” designation identifies counties for which >25% of those aged 25–64 y do not have a high school diploma or equivalent, based on the 2000 census [32]. Because the local prevalence of college graduates is the standard measure of workforce human capital used by economic geographers, we also used the proportion of people aged 25 y and over who had completed 4 y of college. For bivariate and multivariable analyses, we divided the sample of hospitals into quartiles on the basis of the prevalence of college graduates in the local county.

For each of the dimensions of locational resources, one resource level was designated “locationally disadvantaged” (persistently poor, high unemployment, entire county designated as HPSA, high prevalence of non-high school graduates in workforce, lowest quartile college educated). Dimensions of advantage/disadvantage were moderately intercorrelated between counties, with unweighted values of Cramer's V ranging from 0.21 (HPSA designation and prevalence of college graduates in the local county) to 0.56 (chronicity of local poverty and high unemployment).

#### Individual hospital characteristics

Measures of hospital size and ownership were derived from the 2003 Medicare Cost Reports. From the same source, the ratio of interns and residents to beds was used to compute a 3-valued measure of teaching status, with a cut point of 0.10 separating “major” and “minor” teaching institutions, and hospitals with a value of zero designated as “non-teaching” [33]. Location was classified using the Office of Management and Budget's 2003 urban/rural continuum codes, collapsed into three standard categories: “metropolitan,” “micropolitan” (town or small city), and “non-core” (roughly, rural). From the Cost Reports we computed the percent of bed days attributable to Medicaid revenue, and total margin in 2003. Cost Report data were unavailable for 175 (6.4%) of the hospitals. Those institutions were excluded in multivariable analysis.

#### Attainment, improvement, and net performance score (Performance Assessment Model)

To assess potential redistribution under VBP, we used the Performance Assessment Model that is detailed in Appendix B of CMS's November 2007 Report to Congress [5]. This model is “the methodology that could be used for scoring hospital performance on specific measures” in hospital VBP, according to CMS's January 2009 *Roadmap for Implementing Value Driven Healthcare in the Traditional Medicare Fee-for-Service Program* [6]. Under the model, hospitals are assigned scaled two scores for each condition annually, one based on attainment (absolute performance) and the other based on improvement (increase in performance from the prior reporting year). A net performance score is then assigned for each condition equal to the attainment or improvement score, whichever is greater.

**Figure 1:**
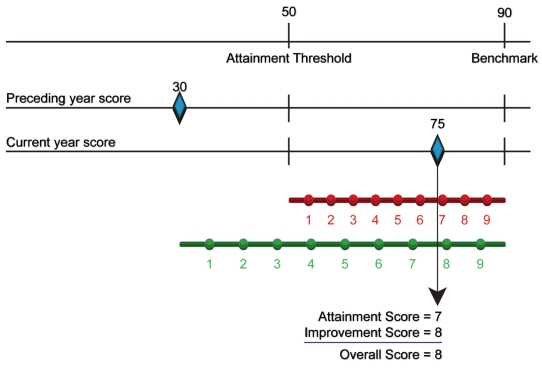
Performance Assessment Model scoring example (standard method). For this example, the attainment threshold is 50 points, and the benchmark is 90 points, based upon hospital performance nationwide during the previous year (see text). This hypothetical hospital received a composite score of 30 during the preceding year, and a score of 75 during the current year. This is converted to attainment and improvement scores, as follows: For the attainment score, the current year score falls in the attainment range, and the hospital is assigned a scaled attainment score of 7. For the improvement score, the current year score falls in the improvement range, and the hospital is assigned a scaled score of 8. The overall score is the larger of the two scores (a value of 8). Figure adapted from the Centers for Medicare and Medicaid Services Report to Congress (2007) [5].

We assigned these scores to hospitals using the model's “standard” method, as illustrated in Figure 1. To determine the attainment score, for each condition, for each year, there is an attainment range with an upper limit benchmark (the mean of the top decile of performance for all hospitals, for the previous year) and a lower limit attainment threshold (the 50th percentile of performance for all hospitals, for that the previous year). Hospitals at or below the attainment threshold receive 0 attainment points. Those at or above the benchmark receive 10 attainment points. Hospitals performing within the attainment range receive a scaled score between 1 and 9 attainment points. A similar scale methodology generates the improvement score, with the upper benchmark fixed at the same level as for that measure's attainment score. However, the lower threshold is defined differently for every hospital, and is set at the hospital's performance score in the previous period. Thus, the improvement scoring range varies across hospitals, and requires a greater increase in the number of points on the part of previous low-performers than previous high-performers in order to obtain the same scaled score. We also assigned scores using the model's alternative method for “topped-out” measures [5]. Results of our analyses were not significantly changed.

### Statistical Analysis

We began by describing the distribution of the sample of hospitals with respect to the individual characteristics and locational resources described above. Then, with hospitals as the units of analysis, we computed mean annual composite HQA performance scores, for each dimension of location, by resource level, and developed 95% confidence intervals (CIs) around each mean. To formally test trends in performance over time, for each condition and dimension, we developed mixed models that included resource level, time, and their interactions as fixed effects, along with random effects of hospital and county, to reflect repeated measures for hospitals and clustering of hospitals within counties. For each condition and dimension, we also assessed differences in mean composite scores, comparing hospitals at the most and the least disadvantaged resource level for that dimension, for the year 2004. These bivariate analyses incorporated robust standard errors to account for the clustering of hospitals within counties. The same set of analyses were conducted for the year 2007.

To assess the independent contributions of the dimensions of location, we developed mixed multivariable models of composite HQA performance score for each condition in 2004 and 2007, entering the locational and hospital characteristics simultaneously as fixed effects, with county as a random effect. We performed regression diagnostics using the approach described by Belsley, Kuh, and Welch [34], and the models performed favorably. Finally, we examined bivariate differences in mean Performance Assessment Model attainment, improvement, and net scores for 2007, for each condition, comparing hospitals at the most and least disadvantaged resource level, within each locational dimension. For these tests, robust standard errors were used to correct for the clustering of hospitals within counties.

We also performed analyses that were weighted for hospital size. Again, the results were quantitatively similar; we present unweighted statistics here.

## Results

Although 4,786 different institutions reported HQA data for at least 1 y during the study period, some did not report certain measures during some years. Of the 3,698 hospitals that reported at least one measure of AMI and HF performance in 2004, 3,147 (85.1%) reported on all seven of the measures used in this study in that year, and of those, 2,705 (85.9%) were “complete reporters,” providing data on all seven measures again in 2007. These 2,705 hospitals formed the cohort for the present study. Compared to all hospitals, complete reporters were disproportionately large, had a teaching mission, and were comparatively advantaged in terms of local economy and workforce. Analysis revealed that hospitals that were complete reporters performed at a higher level than noncomplete reporters, within every stratum of the five dimensions of advantage/disadvantage. We performed sensitivity analyses to assess the extent to which the inclusion criteria may have impacted our findings, as reported below.

For most dimensions of locational resources, relatively few of the sample hospitals are in counties with the lowest resource levels (Table 1). For example, only 130 (4.8%) of hospitals are in counties that have been persistently poor in the years 1970–2000, and 231 (8.5%) are in counties with high unemployment. Nevertheless, for some dimensions, a substantial proportion of hospitals are in relatively disadvantaged locations. For instance, 12.5% of hospitals are in areas with a low prevalence of high school graduates, and nearly 25% of hospitals are in areas where fewer than 16.2% of local adults are college graduates. In all, 873 (32.7%) of the hospitals in the sample are in a county that is locationally disadvantaged on at least one dimension.

**Table 1:** US hospitals reporting on all seven HQA measures from 2004 through 2007, according to individual characteristics and locational resource levels.

Category	Level	*n* (%)
**Individual characteristics**		
**Number of beds**	<100 beds	560 (21.2)
	100–200 beds	866 (32.8)
	>200 beds	1,214 (50.0)
**Ownership**	Government	400 (15.0)
	For profit	435 (16.2)
	Voluntary	1,837 (68.7)
**Teaching status**	Not a teaching hospital	1,675 (63.5)
	Minor teaching hospital	494 (18.7)
	Major teaching hospital	471 (17.8)
**Urban/rural continuum status**	Non-core	334 (12.3)
	Micropolitan	331 (12.2)
	Metropolitan	2,040 (74.4)
**Dependence on Medicaid revenue (2003)**	<6.60%	658 (25.3)
	6.60%–11.99%	655 (25.2)
	11.99%–18.76%	651 (25.0)
	>18.76%	635 (24.4)
**Total margin (2003)**	<−0.7	539 (21.6)
	−0.7 to >2.7	641 (25.7)
	>2.7 to >6.8	642 (26.2)
	>6.8	660 (26.5)
**Locational resources**		
**Chronicity of poverty (1970–2000)**	Persistently poor	130 (4.8)
	Intermittently poor	530 (19.5)
	Never poor	2,045 (75.6)
**Extent of unemployment**	High	231 (8.5)
	Not high	2,474 (91.5)
**Health professional shortage**	Entire county	81 (3.0)
	Portion of county	1,762 (65.1)
	No portion of county	862 (31.9)
**Non-high school graduates in workforce**	High prevalence	338 (12.5)
	Not high prevalence	2,367 (87.5)
**College graduates in workforce**	<16.2% college graduates	669 (24.7)
	16.2–22.7% college graduates	673 (24.8)
	22.7–28.0% college graduates	658 (24.3)
	>28.0% college graduates	705 (26.1)
**Total**		2,705 (100)

### Trends in Performance

**Figure 2:**
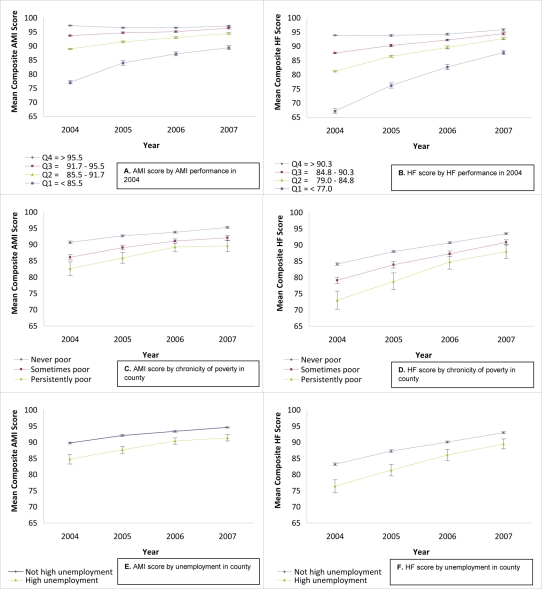
Mean composite HQA performance scores (AMI and HF) for US hospitals, 2004–2007, stratified by various characteristics. Graphics on the left-hand side (A,C,E) reflect temporal trends in composite performance scores for AMI, and those on the right hand side (B,D,F) reflect temporal trends in composite performance scores for HF. (A and B) show mean composite performance score, stratified by performance quartile in 2004. (C and D) show mean composite performance score, stratified by chronicity of poverty in county. (E and F) show mean composite performance score, stratified by unemployment in county. In (A–F), mean composite scores are presented with 95% CIs.

**Figure 3:**
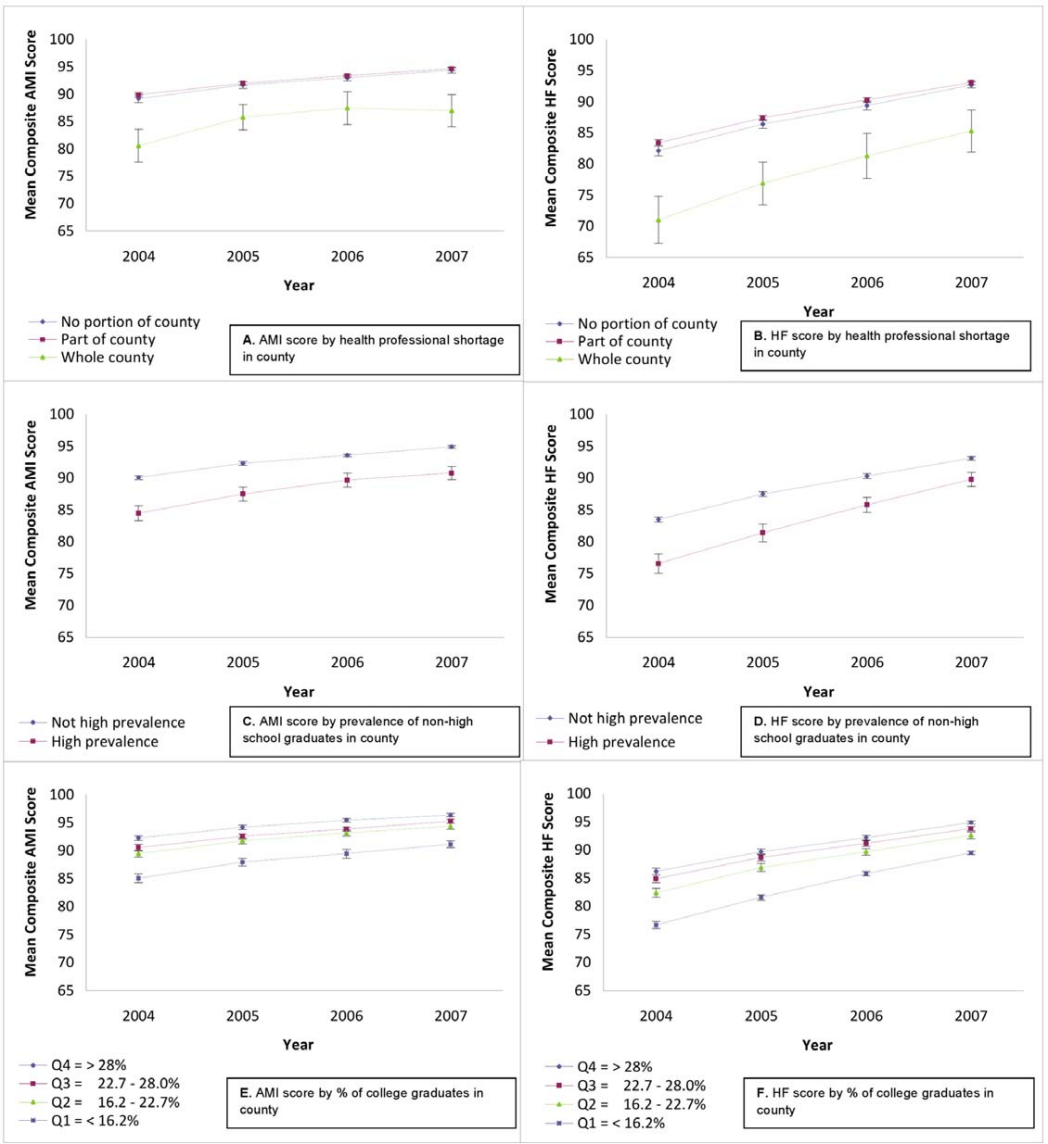
Mean composite HQA performance scores (AMI and HF) for US hospitals, 2004–2007, stratified by various characteristics. Figure 3 continues the series begun in Figure 2. Again, graphics on the left-hand side (A,C,E) reflect temporal trends in composite performance scores for AMI, and those on the right-hand side (B,D,F) reflect temporal trends in composite performance scores for HF. (A and B) show mean composite performance score, stratified by health professional shortage in county. (C and D) show mean composite performance score, stratified by prevalence of non-high school graduates in county. (E and F) show mean composite performance score, stratified by percent of college graduates in county. In (A–F), mean composite scores are presented with 95% CIs.

There was general improvement in mean composite score over time (for all hospitals, for AMI, 1.62 points/year, 95% CI = 1.55–1.70; for HF, 3.33 points/year, 95% CI = 3.24–3.40).Details of the yearly trends in composite scores for the two conditions are depicted in Figures 2 and 3. The top panels in Figure 2 (Figure 2A and 2B) shows trends defined by baseline (2004) performance quartile. For both conditions, hospitals starting in the lowest quartile showed the most improvement over time (*p*<0.001 for a comparison of the linear time trend between the first and fourth quartiles, for both conditions), but even by the fourth year of reporting, those in the initial lowest quartile had not reached parity with the other groups (*p*<0.001 for the difference of means for the first and fourth quartile in 2007, for both conditions). The remaining panels in Figures 2 and 3 display performance trends over time for the five dimensions of locational resources, stratified by resource level. For all five dimensions, hospitals at the most disadvantaged level of resources fared relatively poorly at the outset (*p*<0.001 for the difference between hospitals at the most and least disadvantaged resource levels for all dimensions, for both conditions), and that significant disadvantage continued, but was attenuated over time (*p*<0.05 for the difference in linear trend between hospitals at the most and least disadvantaged resource levels, for all dimensions, for both conditions, with the exception of health professional shortage for AMI). By the fourth year of performance reporting, hospitals in disadvantaged areas continued to lag significantly behind their advantaged counterparts (*p*<0.001 for the difference in means between hospitals at the least and most disadvantaged resource levels for all dimensions, for both conditions) (Table S1).

### Multivariable Analysis of Dimensions of Location

The five dimensions of location are conceptually interrelated, and may be correlated with individual hospital characteristics. Table 2 shows the change in composite performance score independently attributable to individual hospital characteristics, and then independently attributable to each locational dimension, expressed as a difference in score between each level and the most locationally advantaged level, within each dimension. The data show consistent independent effects for chronicity of poverty, entire county health professional shortages, and percent college graduates, with levels of chronicity of poverty and percent college graduates in the workforce showing dose-response relationships to performance.

**Table 2:** Independent effects of individual characteristics and locational resource levels on hospital composite performance scores for AMI and HF, 2004 and 2007.

Category	Level	AMI		HF	
		2004	2007	2004	2007
**Individual hospital characteristics**					
**Number of beds**	<100 beds	—	—	—	—
	100–200 beds	1.7**	1.7**	1.7**	1.8**
	>200 beds	3.8**	3.3**	2.9**	3.0**
**Ownership**	Government	—	—	—	—
	For profit	−0.9	0.9**	−1.8**	0.7
	Voluntary	0.7	1.0**	0.5	0.7*
**Teaching status**	Not a teaching hospital	—	—	—	—
	Minor teaching hospital	1.0**	0.3	0.8	0.5
	Major teaching hospital	2.8**	1.4**	3.8**	1.3**
**Urban/rural continuum status**	Non-core	0.2	−0.4	−4.8**	−2.5**
	Micropolitan	−0.9	−0.5	−2.3**	−0.2
	Metropolitan	—	—	—	—
**Medicaid revenue dependence (2003)**	<6.60%	0.8	0.6*	0.7	0.5
	6.60%–11.99%	0.9*	0.7*	1.1*	0.6
	11.99%–18.76%	1.1*	0.2	0.7	0.5
	>18.76%	—	—	—	—
**Total margin (2003)**	<−0.7	—	—	—	—
	−0.7 to >2.7	0.9*	0.9**	0.8	0.4
	>2.7 to >6.8	2.3**	1.1**	2.0**	0.8*
	>6.8	2.0**	1.3**	2.0**	0.6
**Locational resources**					
**Chronicity of poverty, (1970–2000)**	Persistently poor	−3.9**	−2.7**	−4.4**	−2.0*
	Intermittently poor	−2.8**	−1.5**	−2.3*	−1.2*
	Never poor	—	—	—	—
**Extent of unemployment**	High	0.7	0.6	−1.3	0.9
	Not high	—	—	—	—
**Health professional shortage**	Entire county	−2.6**	−2.7*	−2.7*	−2.2*
	Portion of county	−0.7	−0.1	0.7	0.2
	No portion of county	—	—	—	—
**Non-high school graduates in workforce**	High prevalence	−1.6**	−1.9**	−1.9*	0.5
	Not high prevalence	—	—	—	—
**College graduates in workforce**	<16.2% college graduates	−3.1**	−1.9**	−3.3**	−1.9**
	16.2%–22.7% college graduates	−1.4**	−1.2**	−1.8	−1.3*
	22.7%–28.0% college graduates	−1.0	−1.0**	−0.2	−0.7
	>28.0% college graduates	—	—	—	—

Statistics are derived from mixed models in which hospital characteristics and locational resources entered simultaneously; see text for details. Omitted (comparison) groups are indicated with dashes.

*coefficient significant with *p*<0.05.

**coefficient significant at *p*<0.01.

### Location and Performance Assessment Model Score

Applying the Performance Assessment Model to the 2007 data allowed calculation of attainment, improvement, and net Performance Assessment Model scores (Table 3). Hospitals in more advantaged locations had substantially higher attainment scores (*p*<0.01 for all five dimensions in both AMI and HF). Hospitals in advantaged locations also had higher improvement scores than their disadvantaged counterparts, though differences in improvement scores were narrower and not all statistically significant (*p*<0.05 for all dimensions for AMI, but only for chronicity of poverty and college graduates in the workforce for HF). For locationally advantaged hospitals, attainment generally exceeded improvement; the converse was true for disadvantaged hospitals. The net result was that the Performance Assessment Model score–the suggested basis for reimbursement under VBP–was consistently higher for hospitals in the most advantaged locations than those in the least advantaged locations, for both conditions and for all five locational characteristics (*p*<0.02 for both conditions, all five characteristics).

**Table 3:** Mean Performance Assessment Model scores (attainment, improvement, and net score), by hospital locational resource levels, US Hospitals, 2007.

Locational Resource	Level	Mean (*p*-Value)							
		AMI				HF			
		Attainment Score	Improvement Score	Difference	Net Score	Attainment Score	Improvement Score	Difference	Net Score
**Chronicity of poverty (1970–2000)**	Persistently poor	1.6 (0.001)	3.0 (0.002)	−1.3 (0.001)	3.6 (0.001)	2.5 (0.001)	3.3 (0.001)	−0.8 (0.001)	4.0 (0.001)
	Intermittently poor	2.6	3.2	−0.6	4.0	3.5	3.9	−0.3	4.7
	Never poor	4.1	3.7	0.4	5.0	4.5	4.3	0.3	5.4
**Extent of unemployment**	High	2.1 (0.001)	2.9 (0.002)	−0.8 (0.001)	3.6 (0.001)	3.1 (0.001)	3.7 (0.053)	−0.6 (0.001)	4.5 (0.001)
	Not high	3.8	3.6	0.3	4.8	4.3	4.1	0.2	5.3
**Health professional shortage**	Entire county	1.7 (0.001)	3.4 (0.023)	−1.5 (0.001)	3.9 (0.004)	3.1 (0.008)	3.8 (0.158)	−0.7 (0.030)	4.4 (0.014)
	Portion of county	3.7	3.5	0.2	4.7	4.2	4.0	0.2	5.1
	No portion of county	3.9	3.8	0.2	5.0	4.4	4.4	0.0	5.4
**Non-high school graduates in workforce**	High prevalence	2.2 (0.001)	3.2 (0.001)	−0.9 (0.001)	3.9 (0.001)	3.0 (0.001)	3.9 (0.081)	−0.8 (0.001)	4.6 (0.001)
	Not high prevalence	3.9	3.7	0.3	4.9	4.4	4.2	0.2	5.3
**College graduates in workforce**	<16.2% college graduates	2.2 (0.001)	3.3 (0.001)	−1.1 (0.001)	4.0 (0.001)	3.1 (0.001)	3.9 (0.001)	−0.7 (0.001)	4.5 (0.001)
	16.2%–22.7% college graduates	3.4	3.4	0.0	4.5	4.0	3.9	0.1	5.0
	22.7%–28.0% college graduates	4.1	3.7	0.4	5.1	4.7	4.3	0.4	5.5
	>28.0% college graduates	5.0	3.9	1.1	5.6	5.1	4.5	0.6	5.8

Details of the calculation of scores under the Performance Assessment Model (attainment, improvement, and net scores) are described in the text. Within each column, within each dimension of locational resource, the *p*-value is derived from a test of the difference in means between hospitals at the most and least disadvantaged levels.

### Sensitivity of Findings to Inclusion Criteria

To assess the sensitivity of our core findings to our “complete reporters throughout” inclusion criteria, we conducted longitudinal (2004–2007) analyses with the larger sample of hospitals that reported completely in 2004 (*n* = 3,147), as well as cross-sectional (2007) analyses with complete reporters for AMI in that year (*n* = 3,074) and HF (*n* = 3,908). In each case the results were substantially equivalent to those reported here.

## Discussion

We found that US hospitals operating in locations with richer economic and human resources attained significantly higher clinical process scores than those located in less advantaged areas during the period 2004–2007. This pattern was evident along several dimensions of the local economy and workforce. Over the study period, hospital performance improved generally, with initially low-performing hospitals showing the greatest increases. Since locationally disadvantaged hospitals were disproportionately low performers initially, they showed more improvement over the 4-y period, and by the end of the study period, disparities by degree of locational advantage had decreased appreciably. Still, by the fourth year of public reporting, locationally disadvantaged hospitals had not achieved scores comparable to their advantaged counterparts.

Our finding of an association between location and performance does not establish causality or specify a mechanism by which the local economy or workforce affect quality. However, it bears emphasis that the association is consistent with research in health management that suggests that effective human and material resources are essential to hospital performance and performance improvement, and that reported performance is only as strong as the weakest link [35]–[40]. Moreover, regardless of the causal history, the fact that better-endowed providers are significantly better performers suggests that pay-for-performance may transfer funds from providers in disadvantaged locations to their better-endowed counterparts. This possibility has international resonance, as is discussed below.

### Limitations

We selected our process measures carefully to ensure that variations in hospital performance are unlikely to reflect differences in the characteristics of patients served by hospitals. As noted previously, performance on the measures is satisfied by a physician's order, as documented by a medical records abstractor. For example, in order for a hospital to have succeeded in satisfying the process measure for “administration of an aspirin upon admission,” there need only be an order in a patient record (or documentation of a contraindication) that is reported to CMS. Successfully meeting these criteria does not depend upon a patient action or compliance, and is plausibly independent of patient characteristics. Indeed, the Medicare Payment Advisory Commission (MedPAC) has discussed this matter, and has concluded that risk adjustment for patient characteristics “is not necessary” for these measures [41]. This recommendation is consistent with a recent study that found that performance on the measures used in our study was not consistently associated with patient race/ethnicity, within hospitals [29].

We would not suggest that location is the sole determinant of performance. On the contrary, we found substantial within-county variation in performance, some of which was correlated with hospital characteristics including size, ownership, teaching status, and financial strength. Accounting for within-location variation is beyond the scope of this paper, although it is the focus of some of our ongoing work. On a note related to locational determinism, we would not suggest that hospitals necessarily hire and promote the best of local talent. However, having a strong talent pool from which to draw upon is likely to contribute to organizational strength, all else being equal.

Our estimates of effect should be interpreted with some caution. In measuring and assigning “location,” we used county as the geographic unit for analysis, because it is the unit for which information about the characteristics of interest is readily available. However, locational characteristics of hospitals are not necessarily fully defined at the county level. For example cross-county commuting is common in some areas, with over 25% of US workers nationwide crossing county boundaries to reach their workplace [42]. To the extent that more educated workers are drawn across county lines in order to work at high-performing hospitals, the associations reported here understate the relationship between workforce characteristics and hospital performance. A related caveat pertains to variations in county size and population. Across the US, counties range in size from 67 to 227,556 square km [43]. Some counties are small rural areas; others are entire metropolitan regions. Within large counties, there is likely to be substantial heterogeneity of workforce and economic characteristics. The assignment of average characteristics in such counties is especially imprecise, and as a result our estimates of the impact of location are again likely to be biased downward. To explore this effect, we replicated our analyses, omitting hospitals in counties with populations greater than 1 million, and then again omitting hospitals in counties with populations greater than 500,000. As the sample was restricted to smaller counties, estimates of locational impacts generally increased (analyses not shown).

Other caveats have to do with representativeness and generalizability. As we noted at the outset, locationally disadvantaged institutions were relatively underrepresented in our sample because they were less likely to report consistently to CMS than their better-resourced counterparts. For example, in our sample 4.8% of hospitals were in “persistently poor” counties, but in the nation at-large that figure is 7.5%. Therefore, while our sensitivity analysis did not suggest bias in our estimates of the impact of locational disadvantage, disadvantage is more prevalent than our sample would suggest. In addition, we used process measures of performance for only two clinical conditions. It remains to be seen whether similar trends and patterns will be found, when data are available for other conditions, or for measures of the outcomes of care.

Finally, while our analyses suggest that Medicare's pay-for-performance will transfer funds from poorly resourced to better off areas, we cannot assign dollar values to the transfers that are likely to occur, for several reasons. First, our data derive from a period of public reporting, and the addition of payment incentives may influence provider behavior beyond those changes induced by public reporting. Second, the specifics of payment remain to be determined by the Congress and the CMS. Critical issues include the percent of revenue to be withheld under the scheme, the extent to which that revenue will be returned to providers (rather than retained by the Medicare program [5],[6]) and the translation of performance scores to dollar amounts (the so-called “exchange function” [5]).

### Policy Implications

With respect to Medicare, CMS has acknowledged that pay-for-performance may inadvertently worsen the lot of hospitals that “consistently face challenges in improving or maintaining their performance” ([5], p. 85). In its *Report to Congress*, the agency has outlined plans to monitor the distribution of funds as pay-for-performance is implemented. If some subsets of hospitals are disadvantaged under the payment reform, then those hospitals could be offered training, site visits, and other forms of technical assistance, the agency has suggested. Our work argues for a more proactive approach. Rethinking the way that performance is assessed could help avoid some of the “reverse Robin Hood” consequences that are foreshadowed by our analysis. As we have noted, in its current published form, the Performance Assessment Model credits improvement conditional on starting point. This means that baseline low-attainers must have a greater absolute score increase in order to “improve” as much as baseline high-attainers. Since locationally disadvantaged hospitals are typically baseline low-attainers, they are perforce less likely to be identified as high performers under the model. Changing the model so that it credits improvement regardless of starting point, or assesses improvement over a longer time frame, could help make the program more equitable. It is important to note that the Performance Assessment Model, while referenced in multiple CMS documents as a likely basis for Medicare reform, is still preliminary. Thus, opportunities exist to modify and improve upon the current version. Alternatively, rather than altering the model, CMS may wish to consider comparing hospitals to their similarly located peers, thereby enhancing equity through an “apples-to-apples” comparison. However this strategy carries the risk of institutionalizing inequalities, and finding the right balance may be difficult [44],[45].

Do our findings apply beyond the US? While our specific measures of locational disadvantage may not apply everywhere, there are likely analogs in other settings. For instance, significant health workforce inequalities can be found within and across nations, around the world [46],[47]. These are likely to translate into regional differences in capacities to perform. A recent study in the UK found that general practitioner practices in deprived areas are disproportionately staffed by older physicians, and those who received their medical training outside of the UK [48]. Both of these provider characteristics were associated with poorer practice performance under a pay-for-performance scheme. Deprived areas had a disproportionate share of the lowest performing general practices. In other words, location was linked to performance among UK general practitioners, perhaps through health workforce inequalities. While this remains to be explored in future research, it implies (as does our work) that the pursuit of efficiency through provider accountability may be at odds with pervasive structural inequalities.

Such inequalities can be addressed through countervailing policies. For example, in the UK, geographically targeted approaches have been taken to overcome regional inequalities under “deprivation payment” schemes [49]. In the US, there is no comprehensive strategy to address regional resource inequalities as they might affect health care delivery, although various policies have supported health care in rural areas and in the so-called “safety net,” which includes institutions providing care to low income people [11],[50],[51].

Despite claims that “the world is flat” [52]—that place is irrelevant in a globally networked world—our work suggests that location is a critical input to health care quality. Holding providers accountable is not an unreasonable approach to quality improvement. However, it must be done in a way that attends to the profound inequalities in local circumstances that shape life in the twenty-first century [14].

## Supporting Information

Table S1 Numeric point estimates shown in Figures 2 and 3.
(0.10 MB DOC)

## Abbreviations

AMI: acute myocardial infarction
CI: confidence interval
CMS: Centers for Medicare and Medicaid Services
HF: heart failure
HPSA: Health Professional Shortage Area
HQA: Hospital Quality Alliance
VBP: Value-Based Purchasing
