# Supplementary material for: Hospital Performance, the Local Economy, and the Local Workforce: Findings from a US National Longitudinal Study
Source: PLoS Med. 2010 Jun 29;7(6):e1000297. doi: 10.1371/journal.pmed.1000297 (PMC2893955; doi:10.1371/journal.pmed.1000297)
Supplement: Table S1 — Numeric point estimates shown in Figures 2 and 3. (0.10 MB DOC) [file pmed.1000297.s001.doc]

**Table S1 - Statistics corresponding to Figures 2 and 3 ***

| **AMI PERFORMANCE SCORE** |  |  |  |  |  |
| --- | --- | --- | --- | --- | --- |
|  |  | **2004** | **2005** | **2006** | **2007** |
| **AMI performance in 2004** | Q4 => 95.5 | 92.3 | 94.2 | 95.5 | 96.4 |
|  | Q3 = 91.7 – 95.5 | 90.6 | 92.6 | 93.9 | 95.3 |
|  | Q2 = 85.5 – 91.7 | 89.5 | 91.8 | 93.2 | 94.4 |
|  | Q1 =< 85.5 | 85.1 | 88.0 | 89.5 | 91.2 |
|  |  |  |  |  |  |
| **Chronicity of poverty in county** | Never poor | 90.7 | 92.7 | 93.8 | 95.3 |
|  | Sometimes poor | 86.1 | 89.1 | 91.1 | 92.1 |
|  | Persistently poor | 82.6 | 85.9 | 89.3 | 89.6 |
|  |  |  |  |  |  |
| **Unemployment in county** | Not high unemployment | 89.8 | 92.1 | 93.4 | 94.6 |
|  | High unemployment | 84.4 | 87.7 | 90.4 | 91.4 |
|  |  |  |  |  |  |
| **Health professional shortage in county** | No portion of county | 89.2 | 91.7 | 93.0 | 94.4 |
|  | Part of county | 89.9 | 92.0 | 93.4 | 94.7 |
|  | Whole county | 80.6 | 85.8 | 87.5 | 87.0 |
|  |  |  |  |  |  |
| **Prevalence of non-high school graduates in county** | Not high prevalence | 90.1 | 92.3 | 93.6 | 94.9 |
|  | High prevalence | 84.5 | 87.5 | 89.7 | 90.8 |
|  |  |  |  |  |  |
| **% of college graduates in county** | Q4 => 28% | 92.3 | 94.2 | 95.5 | 96.4 |
|  | Q3 = 22.7 – 28.0% | 90.6 | 92.6 | 93.9 | 95.3 |
|  | Q2 = 16.2 – 22.7% | 89.5 | 91.8 | 93.2 | 94.4 |
|  | Q1 =< 16.2% | 85.1 | 88.0 | 89.5 | 91.2 |
| **HF PERFORMANCE SCORE** |  |  |  |  |  |
|  |  |  |  |  |  |
| **HF performance in 2004** | Q4 => 90.3 | 93.9 | 93.8 | 94.3 | 95.8 |
|  | Q3 = 84.8 – 90.3 | 87.6 | 90.3 | 92.2 | 94.4 |
|  | Q2 = 79.0 – 84.8 | 81.2 | 86.5 | 89.6 | 92.7 |
|  | Q1 =< 79.0 | 67.2 | 76.2 | 82.7 | 87.8 |
|  |  |  |  |  |  |
| **Chronicity of poverty in county** | Never poor | 84.1 | 88.0 | 90.7 | 93.5 |
|  | Sometimes poor | 79.1 | 83.9 | 87.3 | 90.8 |
|  | Persistently poor | 73.0 | 78.8 | 84.8 | 88.0 |
|  |  |  |  |  |  |
| **Unemployment in county** | Not high unemployment | 83.2 | 87.3 | 90.1 | 93.0 |
|  | High unemployment | 76.4 | 81.4 | 86.1 | 89.5 |
|  |  |  |  |  |  |
| **Health professional shortage in county** | No portion of county | 82.1 | 86.4 | 89.4 | 92.7 |
|  | Part of county | 83.4 | 87.4 | 90.3 | 93.1 |
|  | Whole county | 71.0 | 76.9 | 81.3 | 85.3 |
|  |  |  |  |  |  |
| **Prevalence of non-high school graduates in county** | Not high prevalence | 83.5 | 87.5 | 90.3 | 93.1 |
|  | High prevalence | 76.6 | 81.4 | 85.5 | 89.8 |
|  |  |  |  |  |  |
| **% of college graduates in county** | Q4 => 28% | 86.2 | 89.7 | 92.2 | 94.9 |
|  | Q3 = 22.7 – 28.0% | 84.9 | 88.7 | 91.2 | 93.8 |
|  | Q2 = 16.2 – 22.7% | 82.4 | 86.9 | 89.7 | 92.6 |
|  | Q1 =< 16.2% | 76.7 | 81.6 | 85.8 | 89.5 |

^Statistics are the point estimates of the mean score within each category for each year.
